# Enhancing the Electrorheological Effect of Crosslinked Poly(ionic liquid)s by Copolymerizing with Acrylonitrile

**DOI:** 10.3390/polym18202582

**Published:** 2026-10-20

**Authors:** Yuchi Liu, Yu Li, Haoming Pang, Jianbo Yin

**Affiliations:** 1 Smart Materials Laboratory, Department of Applied Physics, School of Physical Science and Technology, Northwestern Polytechnical University, Xi’an 710129, China; 2020100440@mail.nwpu.edu.cn (Y.L.); liyu1004@mail.nwpu.edu.cn (Y.L.); panghm@nwpu.edu.cn (H.P.); 2 Shaanxi Basic Discipline (Liquid Physics) Research Center, School of Physical Science and Technology, Northwestern Polytechnical University, Xi’an 710129, China

**Keywords:** poly(ionic liquid)s, acrylonitrile, crosslinking, copolymerization, ER effect

## Abstract

Poly(ionic liquid)s (PILs) are polyelectrolytes that can produce a strong anhydrous electrorheological (ER) effect, which overcomes the limitation of the hydrous ER effect of classic polyelectrolytes. Crosslinking PILs can further broaden the working temperature range of the ER effect of PILs by increasing or eliminating the glass transition temperature. However, the crosslinked network also easily restricts the ion movement and decreases the interfacial polarization and ER effect of PILs. In this paper, we propose a chemical way to enhance the ion movement and ER effect through the copolymerization of crosslinked PILs with polar acrylonitrile (AN). The structure of the obtained copolymers was characterized by different techniques; the dielectric properties of the copolymer particles in suspensions were analyzed by dielectric spectroscopy, and the ER effect of suspensions of copolymer particles was measured by a rheometer under electric fields. It showed that the copolymerization of crosslinked PILs with an appropriate small amount of AN could enhance the ER effect, which originates from the enhancement of the interfacial polarization rate of crosslinked PILs due to the decrease in the activation energy of ions by polar AN copolymerization.

## 1. Introduction

Electrorheological (ER) fluid is a suspension formed by dielectric particles dispersed in insulating oil. Under applied electric fields, an ER fluid can realize a liquid–solid transition quickly and reversibly [1,2,3]. Due to this characteristic, an ER fluid can be used in electro-controllable actuators, dampers, polishing, and so on [4,5,6]. Currently, research on ER fluids focuses on the preparation of dielectric particle materials with high ER activity. Among various materials, polyelectrolytes attracted significant attention due to their strong ER effect, which stems from the interfacial polarization of mobile ion charge carriers within the particles under electric fields [7]. The early polyelectrolyte-based ER materials were based on classic polyelectrolytes [7,8,9,10,11]. The mobile ions in these materials are small size metal cations, such as Li^+^, Na^+^, and K^+^, and the ion pairs have strong Coulombic interactions, so they need to absorb a certain amount of water to activate the ER effect; they belong to a hydrous ER fluid, showing disadvantages including a large leakage current density and water evaporation at high temperature. To overcome the limitation of classic polyelectrolytes, researchers developed ER materials based on polymer electrolytes [12,13,14]. In the polymer electrolytes, metal ions form complexes with oxygen atoms of polyesters to replace the ion pairs. Thus, the metal ions can dissociate to move and induce interfacial polarization in the dry state. However, the leakage current density of the polymer electrolyte-based ER system is still large because of the hydrophilic nature of polyesters.

Recently developed poly(ionic liquid) (PIL)-based ER materials open a new door to prepare anhydrous polyelectrolyte-based ER fluids [15]. PILs are a new class of polyelectrolytes containing large-size ion pairs that can weaken the association energy between anions and cations. Thus, in the dry state, the ion pairs in PILs can be dissociated to enable ion movement [15]. At the same time, some PILs contain large-size fluoric ion pairs, which enable the PILs to have a hydrophobic nature [16]. Due to these characteristics, PILs can act as excellent anhydrous polyelectrolyte-based ER materials, which overcome the limitation of the hydrous ER effect of classic polyelectrolytes. In addition, through designing different side chain structures and selecting different mobile ions, the ion movement and polarization of PILs can be facially adjusted. It has been found that the PILs with relatively flexible segments and strong plasticity (e.g., P[MTMA][TFSI], P[VBTMA][TFSI], etc.) have the maximum room-temperature ER effect [15,17]. However, the glass transition temperature of these PILs is also relatively low, which limits the working temperature range of the ER effect.

Introducing a crosslinked network to restrict the movement of segments could eliminate the glass transition process of linear PILs [18]. However, the network of crosslinked PILs (CPILs) conversely inhibits ion movement within the CPILs. In our previous work, we improved the dielectric constant of several kinds of CPILs via physical absorption of a polar aniline oligomer by the swelling method [19]. We found that absorbing a polar aniline oligomer could increase the dielectric intensity and decrease the activation energy of interfacial polarization of the CPILs with a relatively loose crosslinking network and improve their ER effect, but absorbing aniline oligomer had no contribution to the interfacial polarization and ER effect of the CPILs with a relatively tight crosslinking network due to the increase in steric effect.

On the other hand, in solid polymer electrolytes, the polarity of the matrix is strongly related to the chain mobility, directly influencing the ionic conductivity [20]. A nitrile group could be introduced into polymers using acrylonitrile (AN), yielding polyacrylonitrile (PAN) [21]. Inspired by this structure, we, in this paper, proposed a chemical way to enhance the ion movement and ER effect through the copolymerization of tightly crosslinked PILs (poly[1,2-divinylbenzyl dimethylamine ethane] [hexafluorophosphate] (P[BVDEA][PF_6_])) with polar AN, named CPIL-co-AN. The structure of CPIL-co-AN was characterized by different techniques: the dielectric property of the CPIL-co-AN in suspensions was analyzed by dielectric spectroscopy, and the ER effect of suspensions of CPIL-co-AN was measured by rheometer under electric fields. It showed that the copolymerization of tightly associated CPILs with an appropriate small amount of AN could decrease the activation energy of dissociation and movement of PF_6_^−^ within CPILs and shorten the relaxation time of interfacial polarization, which largely enhance the electro-responsive ER effect of CPILs.

## 2. Materials and Methods

### 2.1. Materials

N,N-dimethylformamide (DMF, AR) and anhydrous ethanol were purchased from Guangdong Guanghua Sci-Tech Co. Ltd. of China. Diethyl ether was purchased from Chengdu Kelong Chemical Co. Ltd. of China. Methanol and 2,2′-azobis(isobutyronitrile) (AIBN, AR) were purchased from Tianjin Fuchen Chemical Reagent Factory of China. 4-Vinylbenzyl chloride (98%, stabilized with TBC) was purchased from Shanghai Macklin Biochemical Technology Co. Ltd. of China. N,N,N,N′-tetramethylethylenediamine (99%), 2,6-di-tert-butyl-4-methylphenol (99.8%), and potassium hexafluorophosphate (K[PF_6_], 99%) were purchased from J&K Scientific Ltd. (Beijing, China). Acrylonitrile (AN, 99%, containing polymerization inhibitor MEHQ) was purchased from Meryer (Shanghai) Biochemical Technology Co. Ltd. of China.

### 2.2. Synthesis of CPIL-co-AN

**Figure 1:**
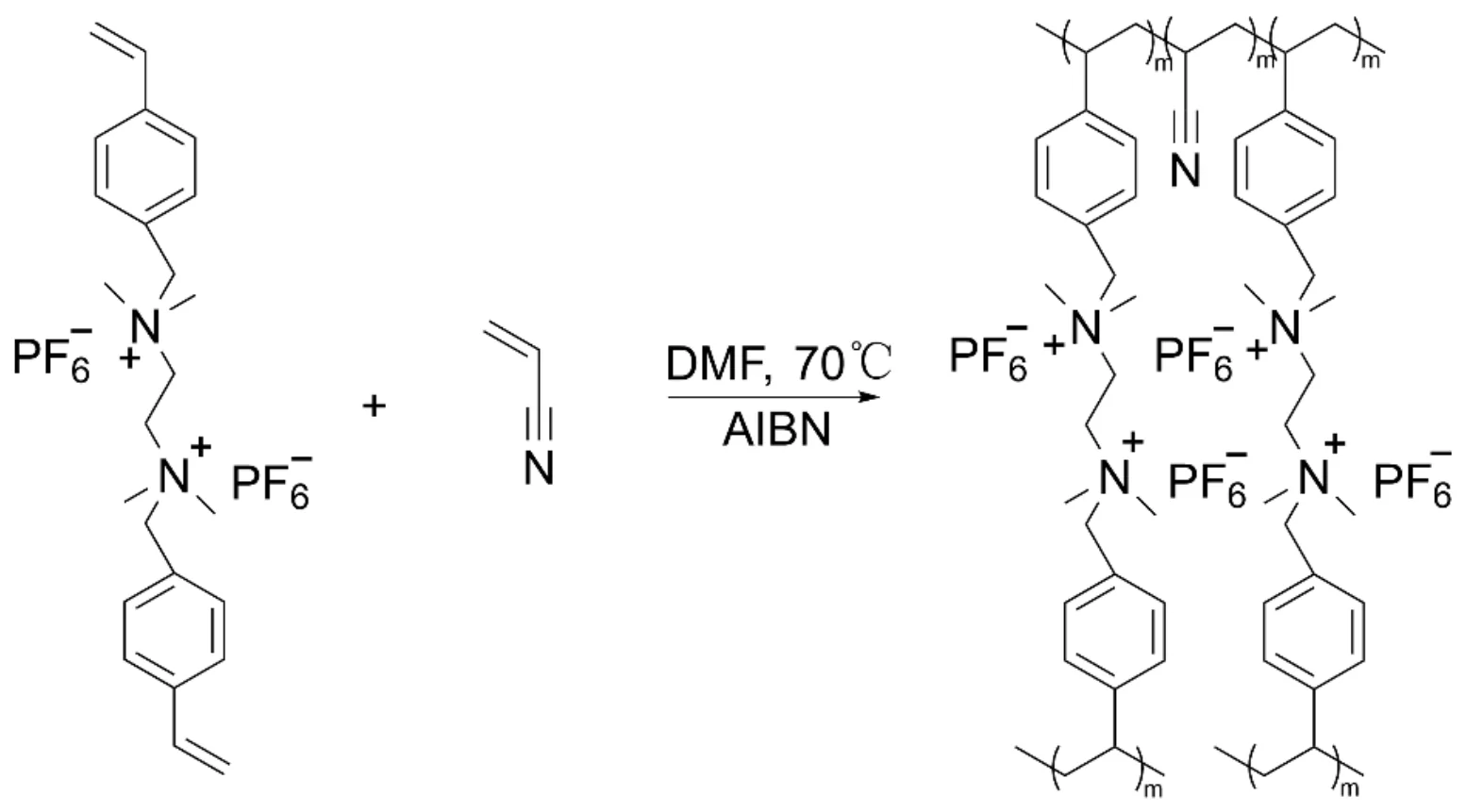
The synthesis process of the CPIL-co-AN.

The synthesis process of the ionic liquid monomer [BVDEA][PF_6_] was described in the previous study [18]. Figure 1 shows the synthesis process of CPIL-co-AN. First, in a 50 mL beaker, [BVDEA][PF_6_], AN, and AIBN as initiator were added into DMF under stirring to form a solution ([BVDEA][PF_6_] (8.453 g, 13.208 mmol), AN (0.010 g, 0.189 mmol), AIBN (0.110 g, 0.671 mmol) and DMF (21.158 g) for CPIL-co-AN-1; [BVDEA][PF_6_] (4.800 g, 7.500 mmol), AN (0.013 g, 0.245 mmol), AIBN (0.064 g, 0.390 mmol) and DMF (12.033 g) for CPIL-co-AN-2; [BVDEA][PF_6_] (3.200 g, 5.000 mmol), AN (0.027 g, 0.509 mmol), AIBN (0.046 g, 0.280 mmol) and DMF (8.068 g) for CPIL-co-AN-3). Then, the solution was transferred into a 100 mL single-mouthed flask. After nitrogen purging for 30 min, the solution was heated to 70 °C and reacted for 24 h. After the reaction was completed, the product was ground and washed with DMF at 100 °C three times. Finally, the product was further washed with anhydrous ethanol at room temperature six times and dried at 80 °C for 24 h to obtain CPIL-co-AN.

### 2.3. Characterization Methods

A Fourier transform infrared (FT-IR) spectrum was measured using a JACSO FT/IR-470 Plus Fourier transform infrared spectrometer (Tokyo, Japan). The thermal decomposition process was measured using a Netzsch STA449F3 thermodynamic analyzer (TGA) (Selb, Germany) in an atmosphere of N_2_, and the temperature was increased from 30 °C to 800 °C at the rate of 10 °C/min. The wide-angle X-ray scattering (WAXS) patterns were measured using a Nano-inXider with the Xenocs SA wide-angle X-ray scattering system (Grenoble, France).

### 2.4. Preparation of ER Suspensions

First, CPIL and CPIL-co-AN were ground and sieved with a 400-mesh sieve to obtain powder-like particles. The density of CPIL is 1.47 g/cm^3^, of CPIL-co-AN-1 is 1.45 g/cm^3^, of CPIL-co-AN-2 is 1.40 g/cm^3^, and of CPIL-co-AN-3 is 1.46 g/cm^3^. Then, the particles were dried and mixed with 50 cSt dimethyl silicone oil at a volume fraction of 20% to obtain the corresponding ER suspensions, and the suspensions were further dried at 120 °C for 48 h in vacuum. Note that there was no penetration/swelling of PIL particles in silicone oil because the PILs used are of high polarity, but silicone oil is nonpolar.

### 2.5. Measurement Methods

The ER effect of ER suspensions was measured by a Thermal-Haake RS600 rheometer (Karlsruhe, Germany) having parallel-plate electrodes with a diameter of 35 mm and a gap of 1 mm. The electric fields were provided by a WYZ-010 DC high-voltage power supply (Beijing, China). The measured temperature was controlled by a circulating oil bath system. The dielectric properties of the ER suspensions were measured by an HP4284A dielectric analyzer (Santa Clara, CA, USA). The ER suspensions were placed in an HP16452A liquid measurement fixture (Santa Clara, CA, USA), and the measured frequency range was 20–10^6^ Hz. The temperature was controlled by a circulating oil bath system within 30–120 °C.

## 3. Results and Discussion

### 3.1. Structural Characterization

**Figure 2:**
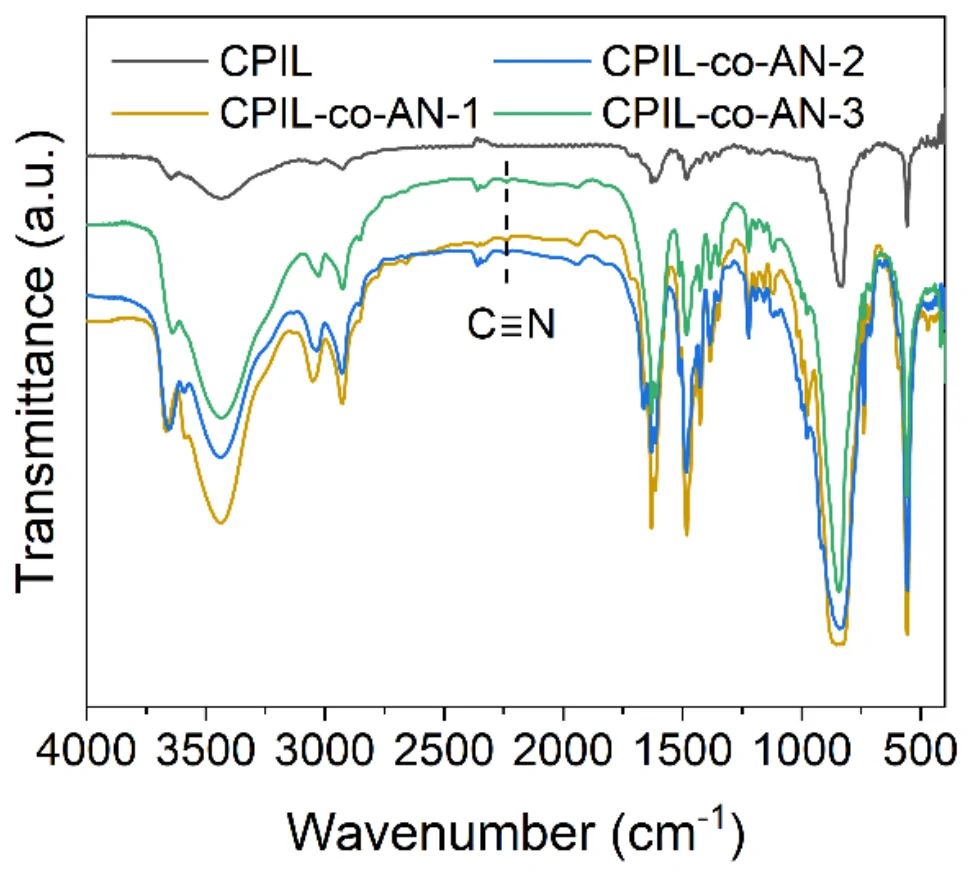
The FT-IR spectra of CPIL and CPIL-co-AN samples.

The chemical structure of the samples was analyzed by FT-IR spectra in Figure 2. For CPIL, the IR characteristic bands belong to -CH_2_-, benzene rings, and quaternary ammonium groups of the polycationic part and the P-F group of the mobile anion PF_6_^−^, as shown in Table 1. For the CPIL-co-AN samples, besides the IR characteristic bands from CPIL, the IR characteristic bands of the cyano group (C≡N) appear, as shown in Table 1, indicating the copolymerization of CPIL with acrylonitrile [22].

**Figure 3:**
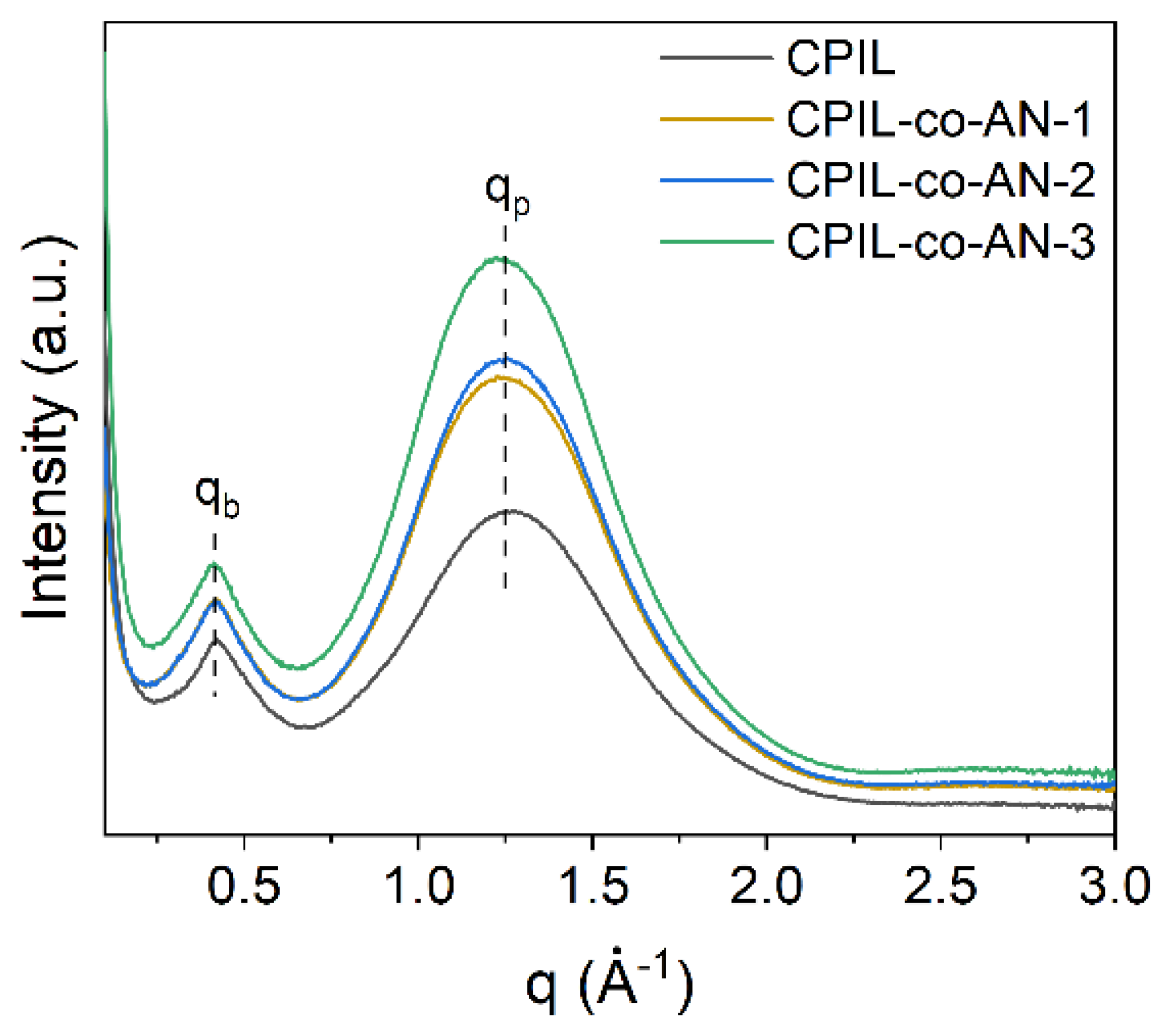
The WAXS spectra of CPIL and CPIL-co-AN samples.

The microstructure of the samples was analyzed by the WAXS patterns in Figure 3. The WAXS pattern of CPIL shows two peaks, where the *q*_b_ peak represents the backbone–backbone correlation distance (*d*_b_) and the *q*_p_ peak represents the pendant group-pendant group correlation distance (*d*_p_) [23]. The correlation distances can be calculated by *d* = 2π/*q*, as shown in Table 2. No *q*_i_ peak corresponding to the ion–ion correlation distance (*d*_i_) is observed, which is consistent with the result for PILs containing PF_6_^−^ counterions [23,24]. After copolymerization with AN, cyanide groups are incorporated into the main chains of CPIL, as shown in Figure 1, and the value of *d*_p_ slightly increases, as shown in Table 2. However, the value of *d*_b_ does not change because the cyanide groups are inserted into the main chains. The cyano group helps the side chains of the CPIL arrangement to be more orderly, so the CPIL-co-AN-3 has the highest *q*_b_ peak. In addition, no new correlation peak due to the phase separation or homopolymerization of the AN domain appears in the WAXS pattern, even if the content of AN is relatively high in CPIL-co-AN-3, indicating the relatively uniform copolymerization of AN units in the CPIL-co-AN sample.

**Figure 4:**
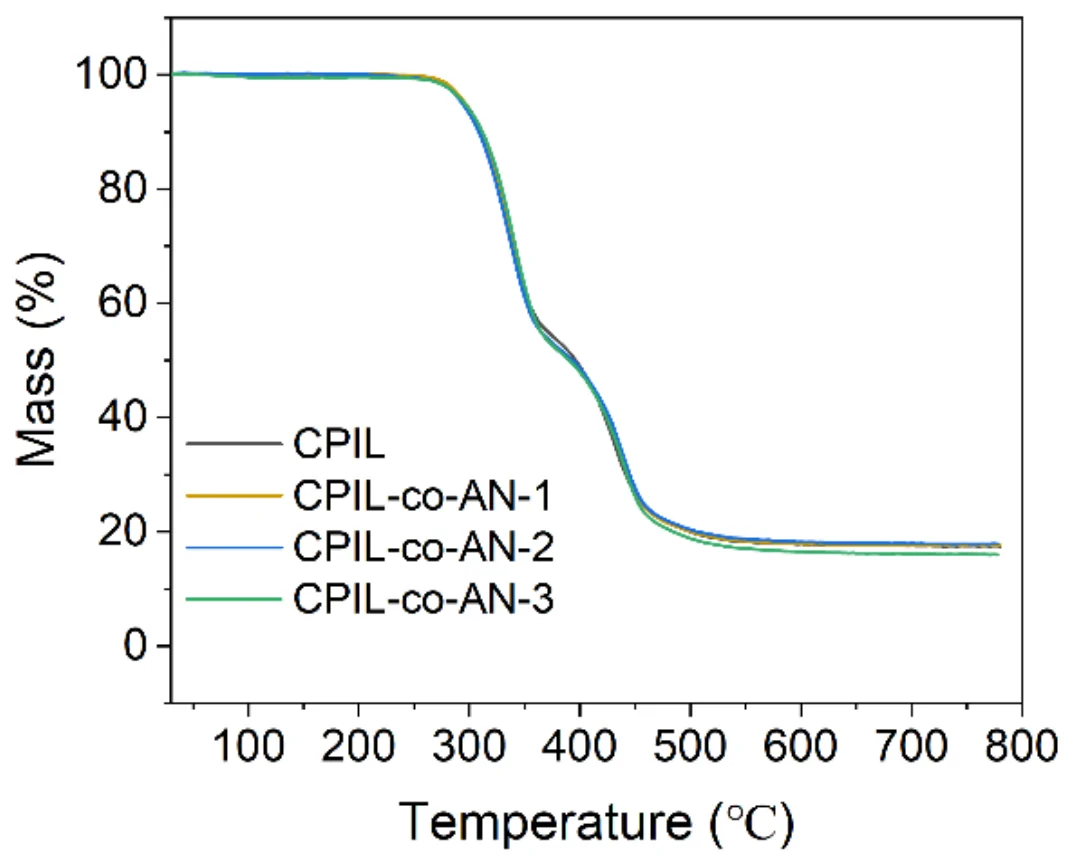
The TGA curves of CPIL and CPIL-co-AN samples.

The thermal properties of samples were measured by TGA in Figure 4. CPIL shows two thermal decomposition stages, which correspond to the polycationic part decomposing between 300 and 380 °C and the PF_6_^−^ counterion part decomposing between 380 and 470 °C. The CPIL-co-AN samples also show two thermal decomposition stages like CPIL, and no additional thermal decomposition stage appears, indicating that no single polyacrylonitrile product is formed. As the AN content increases, the mass loss of the polycationic part slightly increases, as shown in Table 3, due to the incorporation of cyanide groups into the main chain of CPIL.

### 3.2. Dielectric Analysis

**Figure 5:**
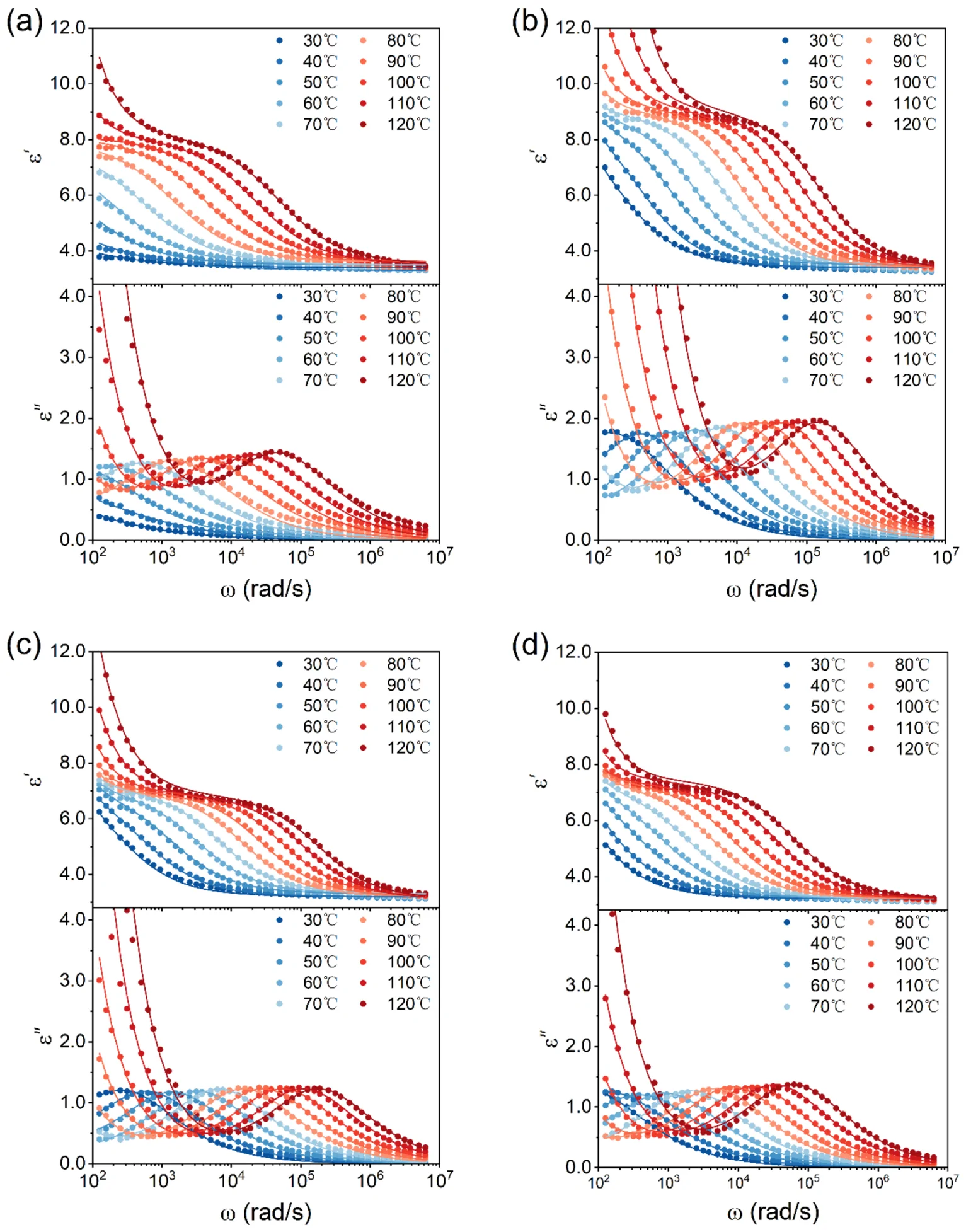
The frequency dependence of dielectric constant *ε*′ and dielectric loss *ε*″ of suspensions containing CPIL (**a**), CPIL-co-AN-1 (**b**), CPIL-co-AN-2 (**c**), and CPIL-co-AN-3 (**d**) at different temperatures. (The solid circle points represent the measured results and the solid lines represent the fitted results with Equation (1)).

The broadband dielectric spectra of the CPIL and CPIL-co-AN suspensions in silicone oil were measured using a dielectric analyzer in Figure 5. It is seen that all suspensions exhibit a dielectric relaxation, which could be attributed to the Maxwell–Wagner interfacial polarization induced by differences in electrical conductivity and dielectric constant between the dispersed particles and the carrier liquid (silicone oil) [25]. As the temperature rises, the relaxation peak gradually emerges and moves to higher frequencies. This indicates a thermal promotion of dissociation and movement of mobile PF_6_^−^ in the CPIL and CPIL-co-AN samples. However, there are significant differences in the relaxation time and relaxation strength among these samples. To further quantitatively compare the relaxation features, we fitted the dielectric data using a Cole–Cole equation incorporated with a conductivity term and an electrode polarization term (Equation (1)) [26]:

(1) $$\varepsilon^{*}\left(\omega \right)=\varepsilon^{\prime }+{i\varepsilon }^{\prime \prime }=\varepsilon_{\infty }^{\prime }+\frac{{∆\varepsilon }^{\prime }}{{1+(i\omega \lambda )}^{\mu }}+i\frac{\sigma_{\mathrm{dc}}}{\varepsilon_{0}\omega }+A\omega^{-b}$$

where ε* represents the complex permittivity of suspensions, while *ω* stands for the angular frequency of the externally applied alternating electric field. The term Δ*ε*′ = *ε*′_0_ − *ε*′_∞_ corresponds to the relaxation strength. Here, *ε*′_0_ and *ε*′_∞_ refer to the low-frequency and high-frequency limiting values of the dielectric constant, which correspond to the frequency ranges below and above the characteristic relaxation frequency, respectively. *λ* = 1/*ω*_max_ denotes the relaxation time, where *ω*_max_ represents the angular frequency at which the dielectric loss *ε*″ reaches its peak value. *μ* is the Cole–Cole dispersion broadening parameter, taking values in the range of 0 < *μ* ≤ 1. *σ*_dc_ is the DC conductivity of suspensions, and *ε*_0_ = 8.854 × 10^−12^ F/m denotes the vacuum permittivity. *Aω*^−*b*^ represents the electrode polarization term.

**Figure 6:**
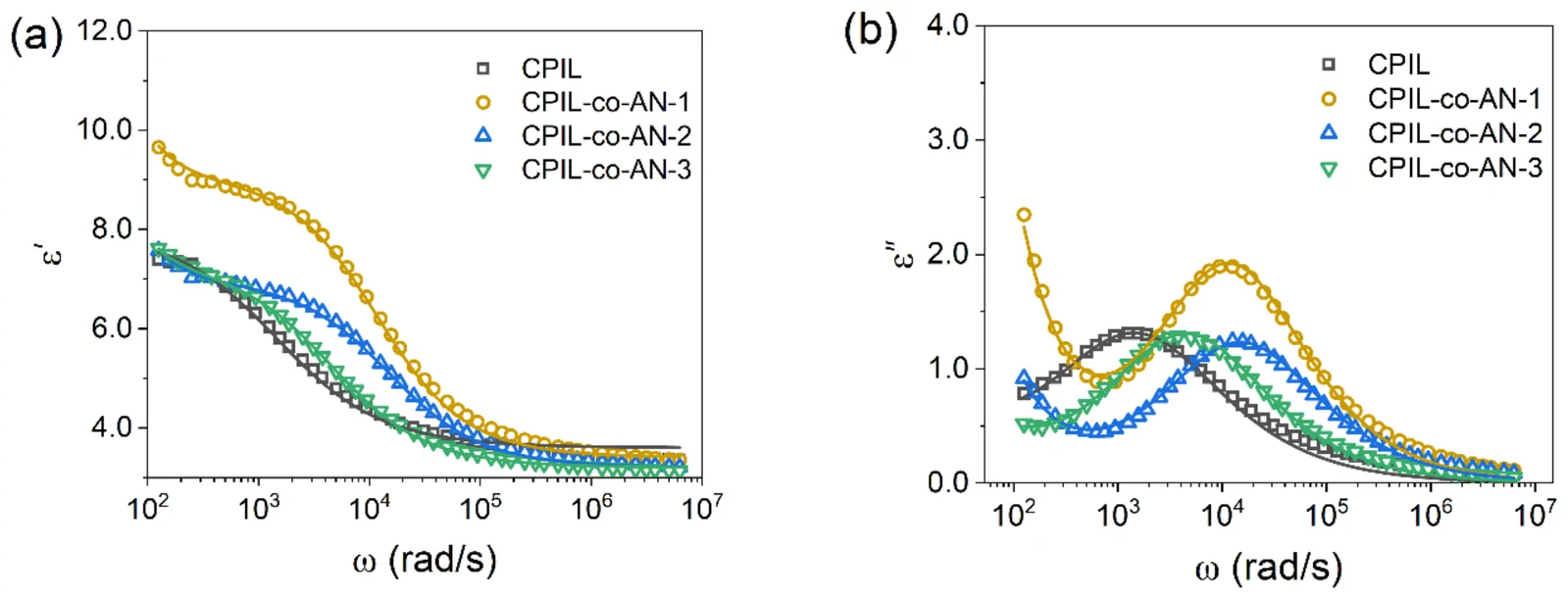
The frequency dependence of dielectric constant *ε*′ (**a**) and dielectric loss *ε*″ (**b**) of suspensions of CPIL and CPIL-co-AN at 80 °C. (The hollow points represent the measured results and the solid lines represent the fitted results with Equation (1)).

The fitting outcomes of the dielectric data for suspensions are presented in Figure 5. Figure 6 shows a comparison of the dielectric data of the suspensions containing CPIL and CPIL-co-AN particles at 80 °C because they show a clear *ε*″ peak. It is seen that the frequency of the *ε*″ peak of CPIL-co-AN suspensions is higher than that of the CPIL suspension, indicating that the CPIL-co-AN samples have a faster relaxation time. But the value of *λ* does not linearly become fast as the AN content increases, and the CPIL-co-AN-2 suspension has the fastest relaxation time as shown in Figure 6 and Table 4. That is, the order of *λ* is *λ*(CPIL) > *λ*(CPIL-co-AN-3) > *λ*(CPIL-co-AN-1) > *λ*(CPIL-co-AN-2). This indicates that copolymerization with an appropriate small amount of polar acrylonitrile can promote the dissociation and movement of PF_6_^−^ within CPIL-co-AN, but too much acrylonitrile does not merit this promotion. In addition, the CPIL-co-AN-1 suspension shows the largest Δ*ε*′ value, but the value of Δ*ε*′ of CPIL-co-AN-2 decreases. This may be because CPIL-co-AN-2 has a higher ionic conductivity and leakage current density. Because there is no appearance of phase separation or homopolymerization of the AN domain according to the characterization above, the fact that the relaxation time becomes slow again in the CPIL-co-AN-3 suspension may be because the relative content of PF_6_^−^ is decreased when the AN content is higher, and thus the ionic conductivity is decreased. Meanwhile, the value of Δ*ε*′ increases again.

**Figure 7:**
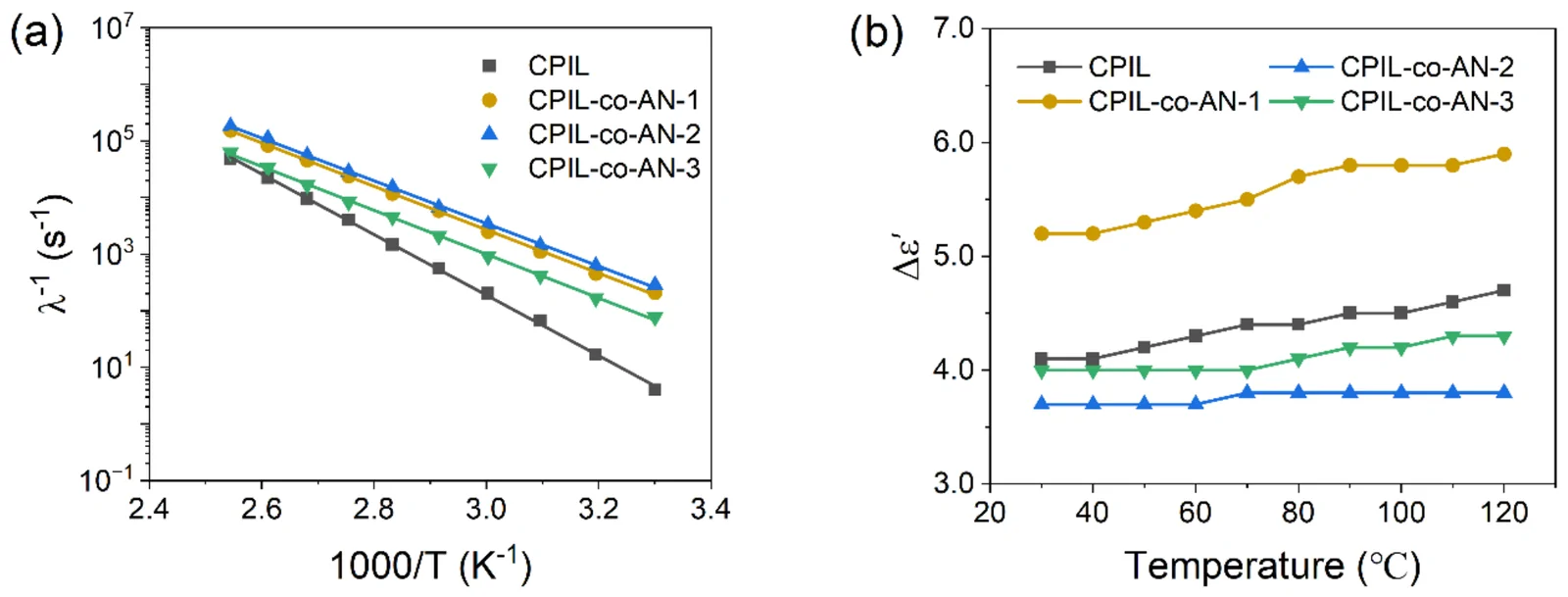
The temperature dependence of (**a**) reciprocal of relaxation time (*λ*^−1^) and (**b**) relaxation strength (Δ*ε*′) of suspensions of CPIL and CPIL-co-AN.

We extracted the values of *λ* and Δ*ε*′, and plotted them with temperature in Figure 7. From Figure 7a, it is seen that the relationship of the reciprocal of relaxation time (*λ*^−1^) and temperature follows the Arrhenius equation (Equation (2)) [27]:

(2) $$\lambda^{-1}=Aexp(-\frac{E_{a}}{RT})$$

where *A* is the fitting parameter, *E*_a_ is the activation energy, *R* is the ideal gas constant, and *T* is the absolute temperature. The value of *E*_a_ for dielectric relaxation of each CPIL-co-AN is lower than that of CPIL, as listed in Table 5. Meanwhile, the value of *E*_a_ for CPIL-co-AN-2 is the lowest. Because the dielectric relaxation of interfacial polarization is closely related to the dissociation and movement of mobile PF_6_^−^, the lower *E*_a_ value means that the copolymerization of an appropriate small amount of AN into CPIL decreases the energy barrier for the dissociation and movement of mobile PF_6_^−^. Two reasons may be attributed to this energy barrier decrease. One is the increase in the dielectric constant of the CPIL matrix after the copolymerization of polar AN. According to the Coulombic equation, $E_{c}=\frac{q_{1}q_{2}}{4\pi \varepsilon_{0}\varepsilon_{p}^{\prime }(r_{1}+r_{2})}$ (where *q*_1_ and *r*_1_ are the charge and radius of mobile ions, *q*_2_ and *r*_2_ are the charge and radius of fixed ions, *ε*′_p_ is the relative permittivity of matrix, *ε*_0_ is the vacuum permittivity) [28], the ion pair interaction energy (*E*_c_) not only depends on the charge number and radius of component ions but also on the dielectric constant of the matrix. Therefore, increasing the dielectric constant of the CPIL matrix decreases the value of *E*_c_. The other reason is the decrease in steric hindrance of the CPIL matrix or the increase in the unopen ‘doorway’ for mobile ions after the copolymerization of polar AN. According to $E_{el}=G_{\infty }4\pi l{\left(r_{1}-r_{D}\right)}^{2}$, where *G_∞_* is the high frequency shear modulus of the matrix, *l* is the ion jump length, and *r*_D_ is an effective radius of an open ‘doorway’ for mobile ions to move from one site to another [29], the elastic potential energy of the matrix (*E*_el_) not only depends on the radius of mobile ions but also on the shear modulus of the matrix. From the WAXS result, it is known that after copolymerization with AN, the pendant group–pendant group correlation distance slightly increases. This reflects the increase in mesh size of the CPIL matrix, which increases the unopen ‘doorway’ for mobile ions or decreases the steric hindrance of mobile ions. Therefore, the value of *E*_el_ of the matrix decreased. Thus, according to *E*_a_ = *E*_c_ + *E*_el_ [30], the value of *E*_a_ is decreased after the copolymerization of AN into CPIL. When the AN content is increased, the value of *l* in CPIL-co-AN-3 increases gradually, so the value of *E*_a_ of CPIL-co-AN-3 is slightly increased.

From Figure 7b, it is seen that the value of Δ*ε*′ of the CPIL-co-AN-1 suspension increases with increasing temperature. This can be attributed to the further promotion of the dissociation and movement of PF_6_^−^ with elevated temperature. The value of Δ*ε*′ of the CPIL-co-AN-2 suspension almost remains constant with increasing temperature. This may be also because CPIL-co-AN-2 has higher ionic conductivity. The value of Δ*ε*′ of the CPIL-co-AN-3 suspension increases with increasing temperature, again because the relative content of PF_6_^−^ is decreased and the corresponding ionic conductivity is decreased in CPIL-co-AN-3.

### 3.3. ER Property

**Figure 8:**
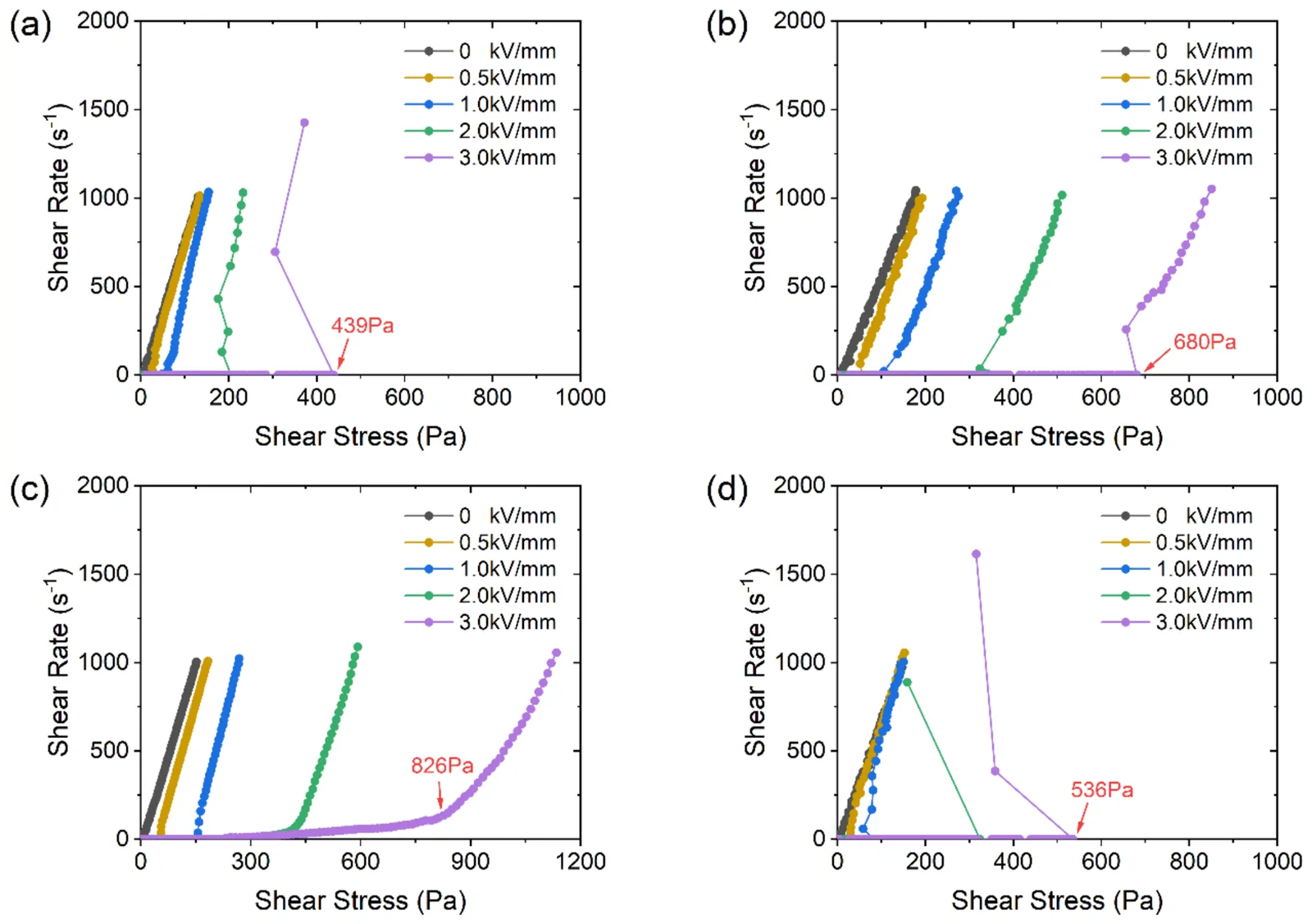
The rheological curves of shear rate vs. shear stress of suspensions measured with the controlled shear stress (CS) mode under different electric fields at 25 °C: (**a**) CPIL, (**b**) CPIL-co-AN-1, (**c**) CPIL-co-AN-2, and (**d**) CPIL-co-AN-3.

Figure 8 shows the rheological curves of suspensions of CPIL and CPIL-co-AN measured with controlled shear stress (CS) mode. Without electric fields, all suspensions start flowing when the shear stress is applied, indicating a liquid-like characteristic. Under electric fields, the suspensions begin to flow when the applied shear stress exceeds a threshold stress. This is because of the particle polarization and the formation of gap-spanning particle chains under electric fields, which hinders the shearing flow. This is the so-called ER effect. The threshold stress is called the static yield stress (*τ*_s_), which is the minimum stress to make suspensions start to flow [2]. The magnitude of *τ*_s_ directly reflects the magnitude of the ER effect. The larger *τ*_s_ is, the stronger the ER effect is. As the electric field strength increases, the values of *τ*_s_ increase due to the increase in electric field-induced interparticle interactions in particle chains. Although the suspensions show the ER effect, the rheogram is significantly different among these suspensions.

The first difference is the magnitude of *τ*_s_. We extracted the values of *τ*_s_ and plotted them as a function of electric field in Figure 10a. It is seen that, at the same field strength, the magnitude of *τ*_s_ consistently follows the order of *τ*_s_(CPIL) < *τ*_s_(CPIL-co-AN-3) < *τ*_s_(CPIL-co-AN-1) < *τ*_s_(CPIL-co-AN-2). This indicates that the ER effect increases after copolymerization with an appropriate small amount of AN. According to the interfacial polarization mechanism of the ER effect [31], Δε′ and *λ* are important to the ER effect because the former is related to the magnitude of interparticle interaction, while the latter is related to the stability of interparticle interaction and the reorganization of particle chains under the simultaneous effect of both electric and shearing fields. It has been proposed that a good ER effect requires the value of *λ* to be located within 10^−3^–10^−6^ s, and the ER effect in this region increases with the reduction in the value of *λ* [31]. From Table 4, the values of *λ*(CPIL-co-AN-1) and *λ*(CPIL-co-AN-2) are in the range of 10^−3^–10^−6^ s, and the values of *λ*(CPIL) and *λ*(CPIL-co-AN-3) are larger than 10^−3^ s. This can explain why the values of *τ*_s_(CPIL-co-AN-1) and *τ*_s_(CPIL-co-AN-2) are higher than *τ*_s_(CPIL) and *τ*_s_(CPIL-co-AN-3). In particular, it is seen that the inverse order of *λ*(CPIL) > *λ*(CPIL-co-AN-3) > *λ*(CPIL-co-AN-1) > *λ*(CPIL-co-AN-2) is consistent with the order of *τ*_s_(CPIL) < *τ*_s_(CPIL-co-AN-3) < *τ*_s_(CPIL-co-AN-1) < *τ*_s_(CPIL-co-AN-2). So, the increase in the ER effect after copolymerization of an appropriate small amount of AN can be attributed to the improvement in interfacial polarization, in particular the shortened relaxation time that merits the stability of interparticle interaction and the rapid reorganization of particle chains [31].

The second difference concerns the yield process. From Figure 8a,d, it is seen that the suspensions of CPIL and CPIL-co-AN-3 show a sudden yielding phenomenon with the rapid increase in shear rate after yield, and then the shear stress largely declines. This indicates that the particle chains are broken by shear, and then they cannot fully reorganize under the simultaneous action of electric and shear fields. From Figure 8b, it is seen that although the suspension of CPIL-co-AN-1 still shows the sudden yielding phenomenon with the rapid increase in shear rate after yield, the shear stress does not largely decline, indicating that the reorganization ability of particle chains becomes good. From Figure 8c, it is seen that the suspension of CPIL-co-AN-2 shows a sudden yielding phenomenon at a low electric field but shows a gradual yielding phenomenon at a high electric field; that is, the shear rate gradually increases, and the shear stress does not decline, indicating that the reorganization ability of particle chains becomes better. It is known that the reorganization ability of particle chains depends on the value of *λ* [31]. According to the yield process analysis, it can be concluded that the order of the reorganization ability is CPIL < CPIL-co-AN-3 < CPIL-co-AN-1 < CPIL-co-AN-2, which is consistent with the inverse order of *λ*(CPIL) > *λ*(CPIL-co-AN-3) > *λ*(CPIL-co-AN-1) > *λ*(CPIL-co-AN-2). Therefore, the increase in reorganization ability after copolymerization of a suitable amount of AN can be attributed to the improvement in interfacial polarization, in particular the shortened relaxation time that merits the stability of interparticle interaction and the rapid reorganization of particle chains.

**Figure 9:**
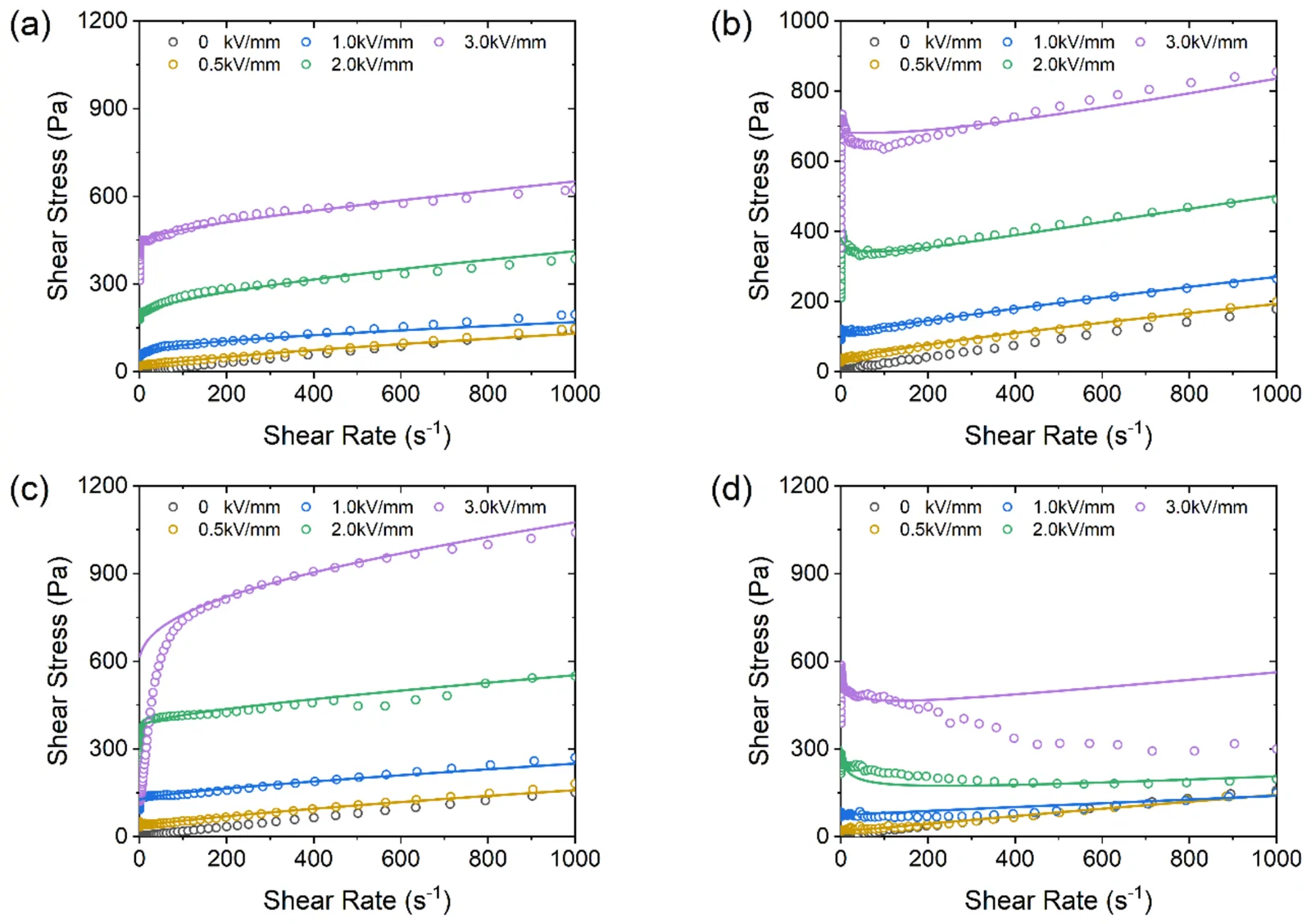
The rheological curves of shear stress vs. shear rate of suspensions measured with the controlled shear rate (CR) mode under different electric fields at 25 °C: (**a**) CPIL, (**b**) CPIL-co-AN-1, (**c**) CPIL-co-AN-2 and (**d**) CPIL-co-AN-3.

Figure 9 shows the rheological curves of suspensions of CPIL and CPIL-co-AN measured in the controlled shear rate (CR) mode. Without electric fields, all suspensions exhibit shear-thinning characteristics with weak yield stress, and the viscosity is low. With electric fields, the shear stress significantly increases, and the suspensions show high yield stress. This is consistent with the result measured in the CS mode because of the particle polarization and the formation of gap-spanning particle chains under electric fields, which hinder the shearing flow. As the electric field strength increases, the value of shear stress increases due to the increase in electric field-induced interparticle interaction in particle chains. From the CR mode rheogram, a significant difference among these suspensions is also observed.

**Figure 10:**
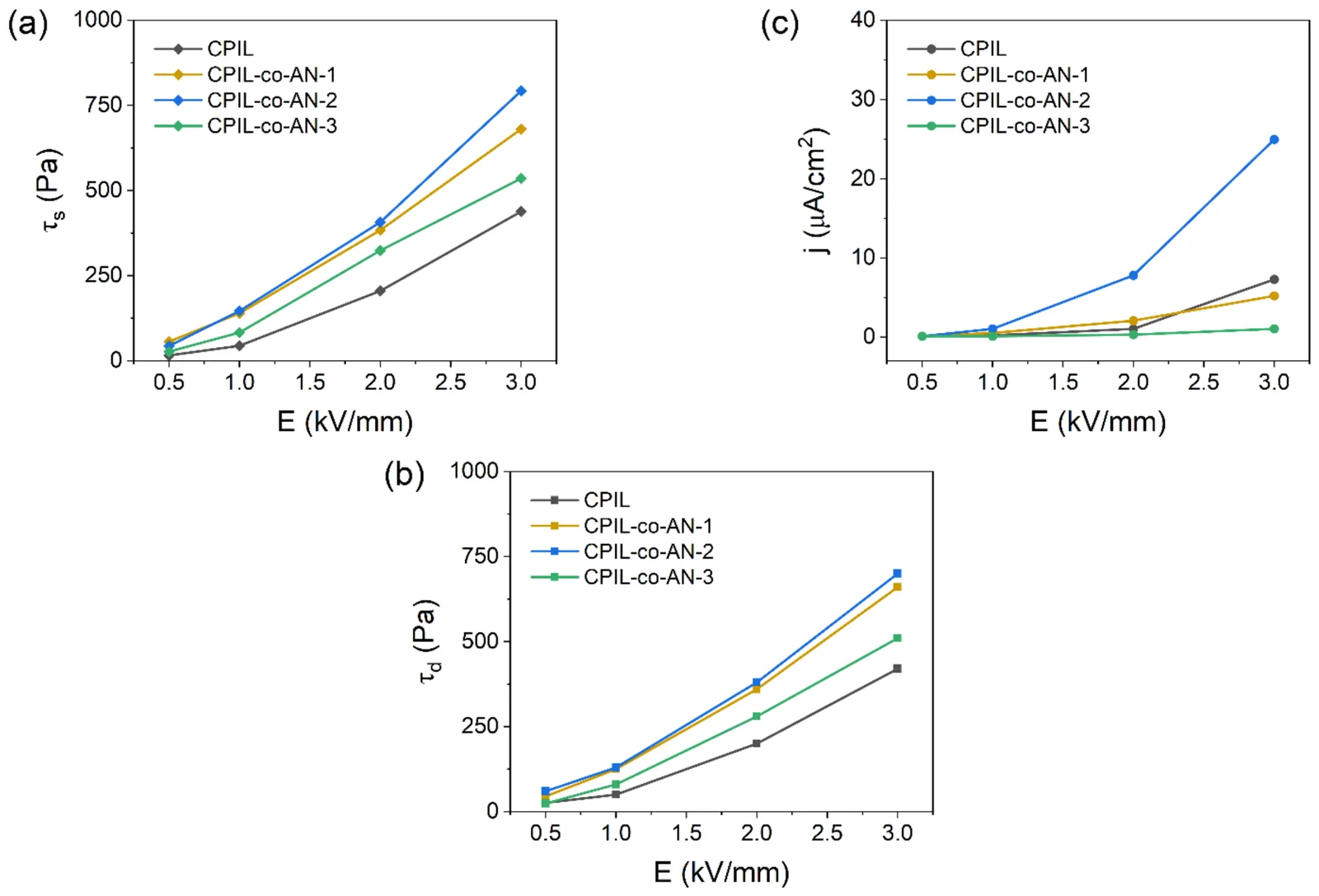
The electric field dependence of (**a**) static yield stress (*τ*_s_), (**b**) dynamic yield stress (*τ*_d_), and (**c**) leakage current density (j) of suspensions CPIL and CPIL-co-AN at 25 °C.

One is the yield stress, namely dynamic yield stress (*τ*_d_), which is the minimum stress to make the suspensions flow continuously. The magnitude of *τ*_d_ also reflects the magnitude of the ER effect. The larger *τ*_d_ is, the stronger the ER effect is. We used the following CCJ equation to extract the values of *τ*_d_ [32]:

(3) $$\tau =\frac{\tau_{d}}{1+(t_{2}\dot{\gamma }{)}^{\alpha }}+\eta_{0}(1+\frac{1}{(t_{3}\dot{\gamma }{)}^{\beta }})\dot{\gamma }$$

where *τ*_d_ is the dynamic yield stress, *η*_0_ is the plastic viscosity at infinite shear rate or at zero electric field, and *t*_2_ and *t*_3_ are time constants used to describe the variation in the shear stress. The exponent *α* is related to the change in the shear stress at the low shear rate region, while *β* in the range 0–1 is for the high shear rate region. At the same volume fraction, a greater *τ*_d_ represents a stronger ER effect. It is seen from Figure 10b, at the same field strength, the magnitude of *τ*_d_ consistently follows the order *τ*_d_(CPIL) < *τ*_d_(CPIL-co-AN-3) < *τ*_d_(CPIL-co-AN-1) < *τ*_d_(CPIL-co-AN-2). It is consistent with the order of *τ*_s_, also indicating that the ER effect increases after copolymerization of a suitable amount of AN. This is also attributed to the increase in reorganization ability after copolymerization of an appropriate small amount of AN due to the shortened relaxation time.

The second difference concerns the flow process after yield. From Figure 9a,d, it is seen that the suspensions of CPIL and CPIL-co-AN-1 and CPIL-co-AN-2 show a stable flow after yield, while the suspension of CPIL-co-AN-3 shows a decrease in shear stress with shear rate after yield. Meanwhile, the flow curve of the suspension of CPIL-co-AN-3 cannot be well fitted by the CCJ equation at high electric fields. This is also attributed to the increase in reorganization ability after copolymerization of an appropriate small amount of AN due to the shortened relaxation time, but too much AN increases the relaxation time and decreases reorganization ability. Thus, at a high electric field, a part of the suspension of CPIL-co-AN-3 is extruded from the gap of electrodes at high shear rate, resulting in a decrease in shear stress. We further extracted the shear viscosity without electric fields (*η*_0_) and with the electric field of 3 kV/mm (*η*_E_) at different shear rates and listed their variation with the AN amount in Table 6. It is seen that *η*_E_ is obviously higher than $\eta_{0}$ and gradually decreases with the increase in shear rate. At $\dot{\gamma }$ = 100 s^−1^ and 1000 s^−1^, the order of *η*_E_ is *η*_E_(CPIL-co-AN-3) < *η*_E_(CPIL) < *η*_E_(CPIL-co-AN-1) < *η*_E_(CPIL-co-AN-2). Although *η*_E_(CPIL-co-AN-3) < *η*_E_(CPIL) is not consistent with *τ*_d_(CPIL) < *τ*_d_(CPIL-co-AN-3), the order still reflects that the ER effect increases after copolymerization of an appropriate small amount of AN. To remove the influence of *η*_0_ on the ER effect, the ER efficiency I was further calculated by *I* = (*η*_E_ − *η*_0_)/*η*_0,_ and its variation with the AN amount is listed in Table 6. With the increase in shear rate, the value of *I* gradually decreases. The order of *I* is also basically consistent with that of *η*_E_, reflecting that the ER effect increases after copolymerization of an appropriate small amount of AN. This is attributed to the increase in reorganization ability after copolymerization of an appropriate small amount of AN due to the shortened relaxation time.

After copolymerization of an appropriate small amount of AN, the current density (*j*) of the suspensions CPIL-co-AN is increased, as shown in Figure 10c. This can be attributed to the decreased activation energy and increased ionic conductivity of CPIL-co-AN with the copolymerization of an appropriate small amount of AN. When the amount of AN is too high, the current density inversely decreases, which is due to the decreased relative content of mobile PF_6_^−^.

## 4. Conclusions

We conducted the copolymerization of tightly crosslinked PILs with polar acrylonitrile and demonstrated that the copolymerization of crosslinked PILs with an appropriate small amount of AN could decrease the activation energy of the dissociation and movement of PF_6_^−^ within CPIL and promote the enhancement of the interfacial polarization rate. As a result, the reorganization ability of CPIL particles was increased, and the electro-responsive ER effect of CPIL was enhanced. Too much acrylonitrile did not merit this ER enhancement because the relative content of PF_6_^−^ was decreased, the corresponding ionic conductivity decreased, and the relaxation time became slow again. The method is simple and can be also used in other cross-linked polyelectrolyte-based ER systems.

## Figures and Tables

**Table 1:** The IR characteristic bands of CPIL and CPIL-co-AN samples.

Sample	IR Bands (cm^−1^)					
	-CH_2_-	Benzene Ring	C-N+	-CH_3_	P-F	C≡N
CPIL	2928	1483, 1631	1222	3031	841	
CPIL-co-AN-1	2927	1483, 1631	1223	3052	846	2237
CPIL-co-AN-2	2930	1485, 1631	1224	3035	842	2240
CPIL-co-AN-3	2926	1484, 1631	1224	3029	845	2239

**Table 2:** The correlation distances in CPIL and CPIL-co-AN samples.

	*q*_b_ (${\dot{A}}^{-1}$)	*d*_b_ (nm)	*q*_p_ (${\dot{A}}^{-1}$)	*d*_p_ (nm)
CPIL	0.421	1.492	1.251	0.502
CPIL-co-AN-1	0.421	1.492	1.225	0.513
CPIL-co-AN-2	0.417	1.507	1.255	0.501
CPIL-co-AN-3	0.413	1.521	1.225	0.513

**Table 3:** The thermal decomposition parameters of CPIL and CPIL-co-AN samples.

Sample	First Step (°C)	Mass (%)	Second Step (°C)	Mass (%)
CPIL	242–377	53.85	377–567	18.13
CPIL-co-AN-1	241–382	51.69	382–570	18.18
CPIL-co-AN-2	251–382	51.83	382–567	18.52
CPIL-co-AN-3	240–381	51.57	381–568	16.82

**Table 4:** The dielectric parameters of suspensions of CPIL and CPIL-co-AN at 30 °C.

Sample	Δ*ε*′	*λ* (s)
CPIL	4.1	2.5 × 10^−1^
CPIL-co-AN-1	5.2	4.8 × 10^−3^
CPIL-co-AN-2	3.7	3.5 × 10^−3^
CPIL-co-AN-3	4.0	1.3 × 10^−2^

**Table 5:** The activation energy (*E*_a_) of relaxation time of suspensions of CPIL and CPIL-co-AN.

Sample	CPIL	CPIL-co-AN-1	CPIL-co-AN-2	CPIL-co-AN-3
*E*_a_ (kJ/mol)	103.37	74.16	72.90	74.90

**Table 6:** The shear viscosity without electric fields (*η*_0_) and with 3 kV/mm electric field (*η*_E_) and the ER efficiency (*I*) of suspensions of CPIL and CPIL-co-AN at 3 kV/mm.

Sample	$\dot{\gamma }$ = 100 s^−1^			$\dot{\gamma }$ = 1000 s^−1^		
	*η*_0_	*η*_E_	*I*	*η*_0_	*η*_E_	*I*
CPIL	0.17	4.64	26.29	0.15	0.62	3.13
CPIL-co-AN-1	0.22	5.82	25.45	0.18	0.86	3.78
CPIL-co-AN-2	0.18	6.74	36.44	0.15	1.04	5.93
CPIL-co-AN-3	0.23	4.21	17.30	0.16	0.30	0.88

## Data Availability

The original contributions presented in this study are included in the article. Further inquiries can be directed to the corresponding author.
